# Robot Transparency and Anthropomorphic Attribute Effects on Human–Robot Interactions

**DOI:** 10.3390/s21175722

**Published:** 2021-08-25

**Authors:** Jianmin Wang, Yujia Liu, Tianyang Yue, Chengji Wang, Jinjing Mao, Yuxi Wang, Fang You

**Affiliations:** 1 Car Interaction Design Lab, College of Arts and Media, Tongji University, Shanghai 201804, China; wangjianmin@tongji.edu.cn (J.W.); michaelyue0812@tongji.edu.cn (T.Y.); laoji@tongji.edu.cn (C.W.); 2133609@tongji.edu.cn (J.M.); wangyuxi@tongji.edu.cn (Y.W.); 2 Shenzhen Research Institute, Sun Yat-Sen University, Shenzhen 518057, China; 3 College of Design and Innovation, Tongji University, Shanghai 200092, China

**Keywords:** on-board robot, robot transparency, anthropomorphism, human–robot interactions

## Abstract

Anthropomorphic robots need to maintain effective and emotive communication with humans as automotive agents to establish and maintain effective human–robot performances and positive human experiences. Previous research has shown that the characteristics of robot communication positively affect human–robot interaction outcomes such as usability, trust, workload, and performance. In this study, we investigated the characteristics of transparency and anthropomorphism in robotic dual-channel communication, encompassing the voice channel (low or high, increasing the amount of information provided by textual information) and the visual channel (low or high, increasing the amount of information provided by expressive information). The results showed the benefits and limitations of increasing the transparency and anthropomorphism, demonstrating the significance of the careful implementation of transparency methods. The limitations and future directions are discussed.

## 1. Introduction

The emergence of on-board intelligent robots has enriched the practical application scenarios for robots, enhanced the human–vehicle interaction experience, and improved the overall intelligence level of the intelligent cockpit. It belongs to the field of Human–Robot Interaction (HRI), which is receiving increasing attention from production and research. The examples of vehicle-mounted robots that have been already used in cars have inspired much academic and industrial research and foresight researches worthwhile in the areas of anthropomorphism, autonomous interaction, and new ways of interacting in self-driving cockpits in the future.

Early robot systems in the 1990s had many limitations, such as accepting only a few simple commands mechanically, having only a fixed set of answers, having no real voice planning or ability to autonomously generate purposeful conversations, etc. [1]. Stimulating interactive robots with human–robot communication capabilities has effectively become an area of active research in the last two decades [1]. Voice communication is one of the main interaction methods to convey information between human and robots, so robots with human-like voice communication capabilities can provide better services. However, natural voice commands do not fully convey precise information, and human sometimes prefer uncertain terms, symbols, and concepts [2].

In the field of on-board robots, a considerable amount of research has proved that anthropomorphic robots provide a better driving experience than traditional forms of interaction with voice and touch screens. For example, Kenton Wiliams et al., from MIT published several papers comparing four different types of interactions (smartphone, dynamic robot, static robot, and human passenger) and showed that dynamic robots had a significant effect on reducing the user’s cognitive load, improving distractions, and spending more time to get a positive affect [3,4]. David R. Large et al. [5] published a study that explored how passengers in self-driving cars interacting with an on-board conversational agent interface, conducting a comparison of three groups of participants with an anthropomorphic agent interlocutor, a voice command interface, and a traditional touch interface. The results showed that the anthropomorphic agent interlocutor was the most popular interface and significantly improved the enjoyment and the sense of control of the journey experience. However, it contained ‘trust challenges’, with participants reporting that they were unable to predict the intentions of the anthropomorphic agent well enough to ensure that they were in control.

Therefore, the key to human–robot interactions is how the human communicates with the robot. Transparency that is well-maintained within the team facilitates is constructive to maintain a proper understanding and sound judgement of intelligent machines and, subsequently, maintain effective collaboration. At the same time, robots are given more life-like expressions by people because of the anthropomorphic nature of their image. Kontogiorgos, Dimosthenis et al. [6] conducted a comparative evaluation study of social robots with anthropomorphic faces versus smart speakers with only voices. The results showed that anthropomorphic expression and communicating with nonverbal cues is not always the best option. Anthropomorphic social behavior needs to be balanced with the task. Therefore, it is also worth considering whether on-board robots should show anthropomorphic aspects of humanity. This study examined the aspects of transparency and their effect on human–robot outcomes when human working together with an on-board robot in driving and nondriving states.

This paper first introduces the theory of human–robot communication transparency and anthropomorphism in Section 2 and discusses how to examine the results on human HRI in Section 3, including safety, usability, workload, trust, and affect. Section 4 presents our evaluation study on communications with on-board robots in driving simulators. Section 5 presents the results of three task scenarios on each evaluation dimension, and the experimental results show that the different transparency information and anthropomorphic human performances of the robot communicate with humans variously. Finally, Section 6 discusses the findings of this study in terms of transparency and anthropomorphism, the suggestion for a human–robot design, and future research directions. In this paper, we contribute to an emerging field by applying a transparent model of human–robot teams and a simple anthropomorphic approach to intelligent robotic communication methods that have been empirically evaluated to inform the design of human–robot interactions.

## 2. Robot-to-Human Communication

One of the important determinants in evaluating whether anthropomorphic robots perform well is maintaining effective communication with humans [7]. It has been shown that effective team member communication, along with other related indicators such as mutual trust and shared mental models, can lead to higher levels of performance [8,9]. Therefore, it is crucial to comprehend the factors that influence effective HRI communication. As an anthropomorphic robot, the dual-channel interaction between voice and vision is essential. With the increasing complexity of the robot system, their ability to communicate grows, resulting in more information exchange. In this paper, we examine aspects of communication-based transparency in anthropomorphic robots in driving and nondriving states and their impact on human–robot team performances, associated cognitive structures, and affective experiences.

### 2.1. Communication Transparency

Transparency can be defined as a way of establishing shared intentions and awareness between human and robotic systems [6]. The key features of transparency in human–robot systems are understanding the robot’s purpose, analyzing their actions, and receiving information from them about decision-making and environment-aware analysis processes. Chen et al. [10,11] regarded transparency as the ability of an intelligent agent (e.g., robot) to effectively communicate with a human being in order to accurately comprehend its current goals, reasoning, and future state. Good transparency in a team facilitates humans to have a proper understanding and sound judgement of intelligent machines and, subsequently, maintain an effective collaboration. Nevertheless, the Situation Awareness-based Agent Transparency model (SAT) proposes three levels of transparency for an autonomous agent, in addition to an explicit sense of the operator’s Situation Awareness (SA): supporting the operator’s understanding of the autonomous agent’s actions and perceptions of the environmental characteristics (SAT1 Level 1); supporting the operator’s understanding of the reasoning behind the autonomous agent’s actions and decisions (SAT2 Level 2); and involving the agent’s predictions of the outcomes of its reasoning, actions, and any uncertainty associated with the information presented (SAT3 Level 3). Starting with L1, L2, and L3, the analysis and selection of good transparency models and their application in the design of robot interactions will help to maintain transparency between human and robots. Furthermore, it will ensure the usability of the intelligent system, the safety and positive emotional experience of the human, and a level of trust in the intelligent system.

### 2.2. Anthropomorphism

People tend to automatically rationalize the behavior of robots when they enter the living space of human being, which is no exception in an automotive space. Apparently, such an anthropomorphic tendency is a powerful driving force for the development of robots [12]. The manifestation of anthropomorphism refers to attributing human motivations, characteristics, and behaviors to inanimate objects, such as speaking technology (as shown in the example described above), and building expectations on this basis [13]. There are many factors that influence robot anthropomorphism, such as movement, verbal communication, emotion, gesture and intelligence, sociocultural background, gender, and group membership [14]. The purpose of robot anthropomorphism research is not only to assess a better experience in experiencing the anthropomorphic tendencies of robots but to also improve the cognitive abilities of intelligent vehicles. People are inspired (to some extent) to believe that this artifact has the ability to think rationally (agency) and feel consciously (experience) [15], which is usually influenced by people’s perception of these human traits and characteristics (voice, social behavior, etc.). In fact, passengers of smart driving vehicles that were anthropomorphized (with human names, genders, and voices) rated their cars’ cognitive abilities higher than those with the same autonomous driving characteristics but without the associated anthropomorphic cues [16]. The purpose of this paper is to explore the relationship between the anthropomorphic perception of voice and vision in transparency and human–robot cooperative team performances.

## 3. HRI Outcomes

### 3.1. Safety

In human–computer interactions, especially in the field of smart cars, the first consideration in human–computer interface design is safety, and all displayed information should meet the requirements of safe driving under the premise of meeting laws and regulations. Therefore, in the case of an increasing number of electronic devices and richer functions in the vehicle, the National Highway Traffic Safety Administration (NHTSA) expressed concern about driving safety and issued Visual-Manual NHTSA Driver Distraction Guidelines for In-Vehicle Electronic Devices guidelines [17] (referencing relevant research in Europe and Japan) that is nonbinding and voluntary, with the intent of guiding designs to reduce driver distractions. In this guideline, the distractions that affect a driver’s focus are divided into three categories: visual distractions, manual distractions, and cognitive distractions. Visual distractions refer to tasks that require the drivers to take their eyes off the road to obtain visual information. manual distractions for tasks that require the driver to take their hands off the steering wheel and operate equipment, and cognitive distractions for tasks that require the driver to take their attention away from the driving task. As people become more tolerant of electronic devices, there may not be such strict restrictions on these, but it is still necessary for on-board robots to consider safety when interacting with people.

### 3.2. Usability 

“Usability is not a quality that exists in any real or absolute meaning. Perhaps it can be best summed up as being a general quality of the appropriateness to a purpose of any particular artefact.” [18] John Brooke’s argument suggests that usability is a fundamental quality that needs to be present for artifacts in general. If the usability is in doubt, especially among robots with concrete images, it is likely that people will stop using it. The feedback from the robot is that it wants to demonstrate different levels of transparency, which may not be consistent with utility, which means transparency is not necessarily proportional to utility. For example, a robot that is too verbose while driving may make it hard for a human to quickly and accurately obtain its intentions, leading to a reduced utility; however, a lack of transparency in some areas may also make the intentions unclear, and the human will make more unnecessary guesses. This study considers whether robots having different information transparency would increase or decrease their utility.

### 3.3. Workload

Workload is a multidimensional concept used to describe the psychological stress or information processing capacity of a person in performing a task that involves mental stress, time pressure, task difficulty, operator ability, effort level, and other factors [19]. Workload is an important construct to HRI, because it can greatly affect the human–robot team performance. It is, in turn, influenced by a variety of factors, such as task structure, performance requirements, human–machine interface, and human individual factors such as experience [20]. Transparency is thought of as having the potential for decreasing and increasing workloads [21,22]. On the one hand, higher levels of transparency can carry useful information for human decision-making; thus, it can reduce the workload or keep the workload unchanged [18,19]. For example, robots can inform people about the cause of abnormal conditions on the road, avoiding them from getting caught in unnecessary guesses and reducing the amount of cognitive computations people need to perform. On the other hand, the extra information provided by the additional transparency might require extra cognitive resources to process. For example, excessively long decision descriptions and overly complex visual animation switching may lead to an increased brain load for humans due to the requirement to process more information. The impact of transparency on the workload may be influenced by a number of different features, such as text for the voice channel and expression animation for the visual channel. Overall, workload is an important cognitive construct to consider when designing transparent communication, as it is helpful to verify what workload range the transparent design is in for humans.

### 3.4. Trust

In the field of human–computer interactions, trust is considered an important factor in the success of human–computer teams [10]. A seminal paper on trust by John D. Lee et al. identified two fundamental components of human–automation cooperation, which are trust and transparency [23]. Trust is a psychological phenomenon, one’s expectation of an outcome or a subjective probability held about the occurrence of a future event [24]. It can also be considered as an attitude coming from impressions of the information provided by the system and past experiences of use. Depending on the information, impressions, and usage experiences, users will develop different levels of trust and, thus, different levels of system dependence. Mui [25] proposed a model based on trust in vehicle automation, which contains three trust dimensions: predictability, dependability, and loyalty. Stedmon et al. [26] showed that people exhibit higher trust and performances when interacting with systems that use human voice communication compared to systems that use synthetic voice communication. Therefore, trust issues also need to be fully considered in human–robot communication teams.

In intelligent driving, adaptive automation can replace the operator’s perceptual capabilities and assist and replace decision-making and action processes [27]. On the one hand, without trust, people may be reluctant to use the system even if it performs well in autonomous driving and lead to its abandonment; on the other hand, too much trust can lead to misuse, i.e., using the system in an unintended way. Under-trust or over-trust caused by these effects can lead to accidents [28]. Therefore, calibrated trust allows one to maintain an ‘appropriate’ level of trust, which is necessary to ensure the optimal distribution of functions among team members and collaboration between man–machine teams. As a result, one of the key elements in establishing the appropriate trust is transparency [29], which indirectly reflects the intelligence of the robot.

### 3.5. Affect

Since human behavior and thought processes are closely related to affects [30], neglecting the user’s emotional state can negatively influence the task performance and trust in the system [31]. It is also applicable to the driving environment. Affect has been used to encompass different constructs, including emotion, feeling, or mood [32]. This study takes affect as an evaluation dimension and indicator and shows that affect influences the attention, perception, and decision-making, which, in turn, influence the driving behavior [33]. Jeon, Walker, and Yim [34] concluded that drivers in a happy or angry moods have decreased driving performances compared to drivers in normal and fearful moods. Negative potency emotions, such as sadness or depression, have also been shown to reduce one’s driving ability [35]. In addition, emotions can be directly related to the driving experience, especially when a person is communicating with a robot while driving (stationary state); this deserves more attention. 

### 3.6. Current Study

**Figure 1:**
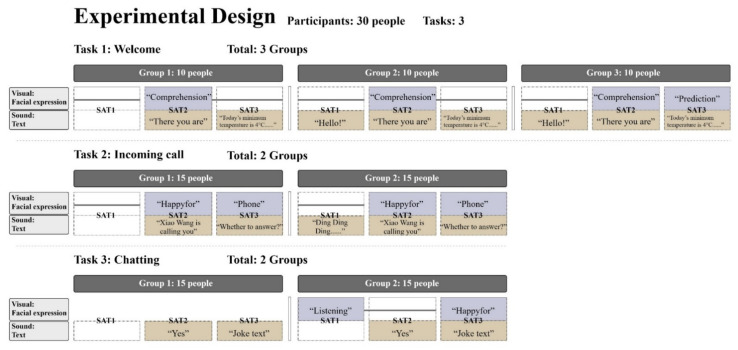
Different information transparency and anthropomorphic designs for the three tasks.

The current study examines the impact of transparency delivered via text messages and team orientation on HRI outcomes (safety, usability, workload, trust, and affect) when working together with an autonomous on-board robot. The conceptual model for the study focus on robot communication transparency will influence the safety, usability, workload, trust, and affect, ultimately leading to improved performances. We investigated the differences between two transparency levels for two tasks in the driving state (whether anthropomorphic visual information and sound information are needed) and three transparency levels for one task in the nondriving state (whether anthropomorphic visual information is required for L3, and whether anthropomorphic L1 sound information is required), sent by the on-board robot to humans via voice and vision (Figure 1).

The L1 sound channel information was chosen to represent the anthropomorphic nature of the robot, because humans usually emit some onomatopoeic words when they perceive their surroundings in life [36,37], and we expect that adding this factor will make the human–robot cooperation a better experience and performance. An intelligent robot generally needs to have L2 understanding and L3 prediction [22]. However, whether all acoustic information is needed with visual information is something to consider; considering the scale of the study, we compared L3 with and without robot expression changes during the stationary task and, while driving, comparing robots with and without expression. We expect that robots with expression changes will have better driving experiences and performances.

## 4. Methods

### 4.1. Participants

Thirty Chinese participants, 25 male and 5 female (22–40 years old, *M* = 28.2, *SD* = 5.83, showing that their ages mainly ranged from 22 to 34), were recruited through questionnaires in this experiment, which contained demographic information about the participants, 83.8% of whom had university or higher education (*n* = 25), followed by junior college (13.3%, *n* = 4), and high school or below education (3.3%, *n* = 1). They all drove more than 2 to 3 times per week, 70% (*n* = 21) used electric vehicles, and 80% (*n* = 24) had some level of knowledge about on-board robots. Informed consent was obtained from each participant prior to data collection. 

### 4.2. Design

There were three tasks in this experiment, and each task was set up with different groups of transparency experiments with different information (Figure 1). Task 1 was a robot under static statues greeting a person who had just boarded a car, and the three groups were set up with different information transparency. When the robot detects and comprehends the person is already on the car, it will send, “Hello! There you are! Good morning.” The robot’s eyes would look at the driver when it said, “There you are!” and winked with a ‘comprehension’ expression. When the robot predicts that the person needs to know the current temperature and dress advice, it will prompt, “Today’s minimum temperature is 4 °C, the weather is getting cooler, pay attention to keep warm!” and make a predictive expression of drinking hot tea. The comparison between Group 1 and Group 2 was whether the L1 anthropomorphic “hello” text message was present or not, while Group 3 added the L1 anthropomorphic “hello” text message and the L3 predicted expression. Thirty participants were equally divided into three groups of ten participants each. After completing the post-SAM affective scale, Task 2 was administered.

Task 2 was a driving robot alerting the driver of a phone call and asking if the driver wanted to answer. Before the start of the task, the participants were told that he or she had a friend named Xiao Wang. While the driver was driving smoothly on a straight road at 30 km/h, the operator-controlled phone rang, and the robot said, “Ding Ding Ding, Xiao Wang is calling you, do you want to answer?” When the robot understood the name of the caller and said, “Xiao Wang is calling you.”, it changed from a smiling foreword expression to ‘happy-for’; when the robot predicted that the person might need to answer the phone, it acted like it was shaking the phone around its ear. The comparison between Group 1 and Group 2 was whether there was an L1 anthropomorphic “Ding Ding Ding” text message. Group 1 and Group 2 of Task 2 had 15 people each and completed the task by filling out the usability, workload, trust, and affect scales, followed by Task 3.

Task 3 was a person chatting with the robot in a driving state. When the person gave the robot the command to tell a joke, the robot made a listening expression at L1, then understood the person’s command, replied, “Yes!”, predicted the content of the joke, and made a ‘happy-for’ expression while the robot was broadcasting. The comparison between Group 1 and Group 2 is that the former one did not have a visual channel for expressions but only had a robot with a shape that can be interacted with by voice. Group 1 and Group 2 of Task 3 also had 15 participants each, and after completing the task, the participants filled out the scales for usability, workload, trust, and affect. This study considered the transparency of different information when humans interacted with the robot by increasing or decreasing the feedback information from the robot in the driving and stationary states from both the visual and auditory channels. The experiment was a between-group experiment, with each participant performing one group from each task, and the number of group tests per task was balanced. 

### 4.3. Apparatus and Materials

**Figure 2:**
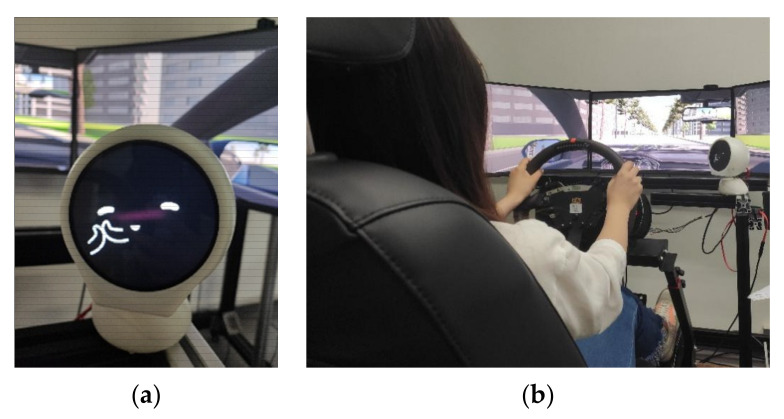
(**a**) Images of the robot ‘XiaoV’. (**b**) Experiment environment.

The prototype on-board robot ‘XiaoV’ (Figure 2a) is an up-and-down structured robot with a head and base. It has a young female voice and can show dynamic videos. XiaoV can move on two levels of freedom, and its head and face will turn to the driver’s seat when interacting with the driver. The feedback from facial expressions and voice output can recommend and describe current events to the drivers. The experimental environment was based on the laboratory’s self-developed car simulation simulator system, and the scenario was developed in Unity software, which simulated a real driving environment. The scenario used in the experiment was a two-way two-lane straight with a large number of oncoming cars in the opposite lane and no overtaking from behind. The simulator was accompanied with a monitoring device, which was used to collect simulator vehicle data and user sweeping behavior data (Figure 2b). All movements of the robot were remotely controlled in real time by the researcher’s laptop (The Wizard of Oz).

#### 4.3.1. Self-Report Metrics

The After-Scenario Questionnaire (ASQ) [38] is a more widely used task-based assessment questionnaire with the benefit of evaluations of three projects: Ease of Task Completion, Time Required to Complete Tasks, and Satisfaction with Support Information, which can be used in similar usability studies. The scale options are set from 1 to 7, with higher scores representing higher levels of performance. The Driving Activity Load Index (DALI) [39] is more appropriate than the other scales for assessing driving-related tasks and contains six dimensions of work—namely, effort of attention, visual demand, auditory demand, temporal demand, interference, and situational stress. The scale options are set from 1 to 10, with higher scores representing a correspondingly higher degree. Mui [25] proposed a model based on trust in vehicle automation, which contains three trust dimensions: predictability, dependability, and loyalty. The present experiment evaluates the trust level from these three dimensions. The scale options are set from 1 to 7, with higher scores representing higher levels. To measure the user (affective) experience, they need to complete the Self-Assessment Manikin (SAM) [40], a nonverbal and pictorial assessment technique used for operationalizing the user experience through the constructs of pleasure, arousal, and dominance. The scale options are set from 1 to 9, with higher scores representing a correspondingly higher degree.

#### 4.3.2. Performance Metrics

In this experiment, the driving data collected in the simulator were used as the evaluation index (Task 1 was performed at rest, without vehicle data). Tasks 2 and Task 3 required the participants to drive smoothly on a straight road at a speed of 30 km/h. The standard deviation of the vehicle speed represents the driver’s longitudinal control ability, and the standard deviation of left lane departure represents the driver’s lateral control ability, examining whether the driver is driving smoothly in the current lane in the lateral longitudinal direction. In the results of Task 2 and Task 3, there were no unsafe situations such as lane crossing, red light running, collision, and so on, as well as no unfinished tasks. Based on such a safe condition, the acceleration labeling difference and the standard deviation of left lane departure were used as the main reference basis, with a small standard deviation being regarded as the safer group. The better group was selected by combining the data of other dimensions comprehensively.

In addition, the total sweep frequency and total sweep duration of the robot were extracted from the video recorded by the simulator. Research suggests that a single sweep that is too long (more than 2 s) is considered to be a driver distraction and may lead to driving safety hazards [41]. In all task results, the raw data of each group were counted, and no participant had a single sweep time longer than 2 s. In addition, the groups with longer and more sweeps showed an increase in the total sweep frequency and total sweep duration at the same time—namely, the total single sweep time did not increase and, consequently, did not pose a threat to the driving safety. In the analysis of the results, we used the sweep data as a reference indicator of the driver’s interest in the robot, similar to the affective dimension. Therefore, the group with a longer and more frequent sweep time was considered in the analysis to have a robot whose performance was more likely to interest the driver in the robot.

### 4.4. Procedure

The entire experiment was conducted on a driving simulator and lasted for about 40 min for each participant. Prior to the start of the study, the participants were given a short period of simulated driving to familiarize themselves with the simulated driving scenario. After completing the test drive, the participants were required to fill out a basic questionnaire, give informed consent, sign a confidentiality agreement, and then begin the experiment with their full consent to record the video. After listening to the task descriptions, the participants were asked to perform the corresponding tasks and complete the scales required for the tasks, and then, the participants were interviewed in a semi-structured manner. The rest was done in the same manner until the experiment was fully completed.

## 5. Results

The data of each experimental group of all the task scenarios are summarized, as shown in Table 1. The experimental evaluation refers to several dimensions from the human–robot outcome in Section 3 above. Therefore, in addition to Task 1 (stationary state), the experimental data of Tasks 2 and 3 are divided into six aspects: Safety, Usability, Workload, Trust, Sweep, and Affect.

### 5.1. Task One: Welcome

#### 5.1.1. Sweep

**Figure 3:**
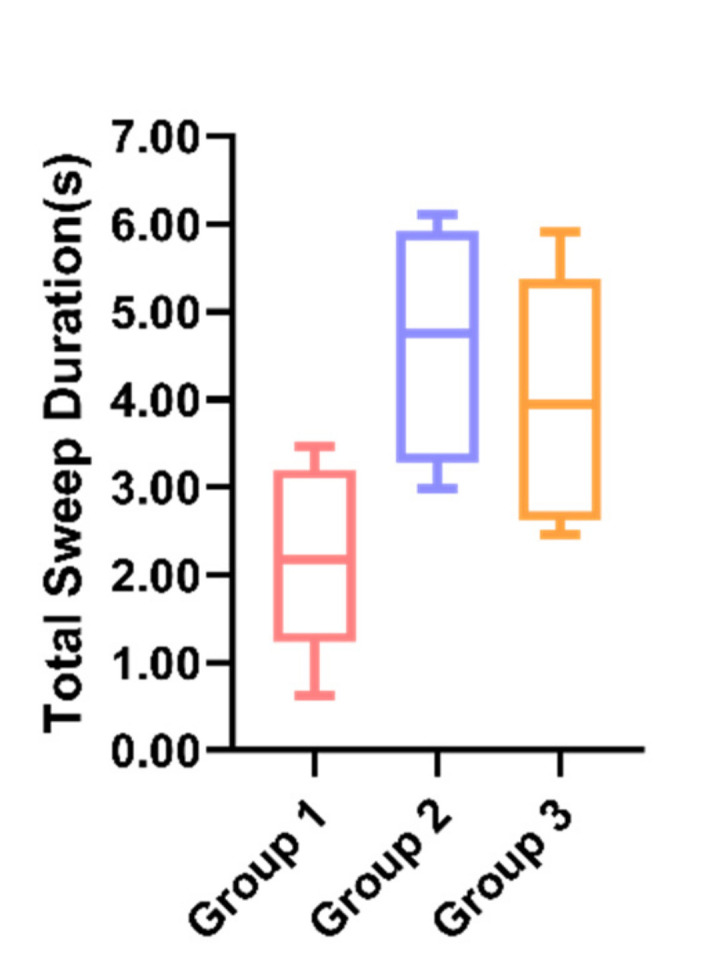
Individual differences in the total sweep duration between the three groups.

In Task 1, a one-way ANOVA was performed on the data from the three groups, whose results showed (Figure 3) that there was a considerable difference in the sweep time between the three groups, *F*(2, 14) = 6.573, *p* = 0.01 < 0.05. A post hoc multiple comparison revealed that the sweep time was significantly less in Group 1 (*M* = 1.97, *SD* = 1.09) than in Group 2 (*M* = 4.65, *SD* = 1.38) and Group 3 (*M* = 4.01, *SD* = 1.50), with no significant difference between Group 2 and Group 3. In Task 1, there was no significant difference in the number of sweeps among the three groups, *F*(2, 13) = 0.116, *p* = 0.89 > 0.05. Therefore, participants in Group 2 and Group 3 had much longer sweep duration means of the robot, which implies that the participants were more interested in the robot performances in Group 2 and Group 3. Additionally, there was no significant difference in the sweep frequency means among the three groups, indicating that the sweep means for the three groups were not markedly different, and the participants in Group 2 and Group 3 had longer single gazes on the robot, i.e., the robot’s performances were more likely to attract the participants’ continuous attention. Since the task was not performed in the case of driving, it can be assumed that Group 2 and Group 3, which elicited longer gazes, had better performances.

#### 5.1.2. Affect

**Figure 4:**
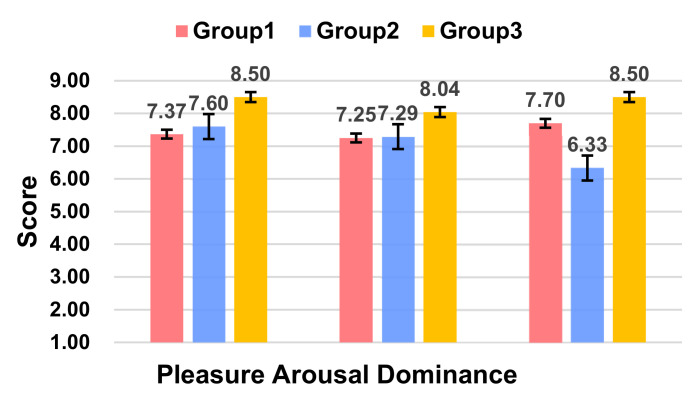
Ratings of Pleasure, Arousal, and Dominance score between the three groups.

According to ANOVA, the participants had higher levels of a pleasure score (*F*(2,20) = 6.529, *p* = 0.007 < 0.05) and dominance score (*F*(2,21) = 3.214, *p* = 0.008 < 0.05). There was no notable difference in activeness (*F*(2,17) = 6.576, *p* = 0.061), but it was close to the *p* = 0.05 significance criterion (Figure 4). A post hoc multiple comparison obtained that Group 3 was significantly better than Group 1 in terms of the pleasure score (*p* = 0.007 < 0.05) and significantly better than Group 2 in terms of the dominance dimensions (*p* = 0.006 < 0.05).

In conclusion, Group 3, which had the anthropomorphic L1 sound information and L3 hierarchical visual information of L1, was able to obtain more attention from the participants, as well as a more positive affective experiences.

### 5.2. Task Two: Incoming Call

#### 5.2.1. Safety

The *t*-test test showed that the average value of the standard deviation of vehicle speed was higher in Group 1 (*M* = 0.42, *SD* = 0.11) than in Group 2 (*M* = 0.27, *SD* = 0.28) in Task 3 but was not significantly different (*p* = 0.30 > 0.05), so the drivers were considered to have essentially the same level of longitudinal vehicle control. In contrast, the standard deviation of left lane departure in Group 1 (*M* = 0.18, *SD* = 0.24) was significantly lower than in Group 2 (*M* = 0.77, *SD* = 0.29) (*p* = 0.000 < 0.001). From the dimension of vehicle safety, it was considered that Group 1, which had a significantly better level of lateral vehicle control, was safer.

#### 5.2.2. Usability

From the usability average scores, it can be concluded that Group 2 (*M* = 6.32, *SD* = 0.68), i.e., with L1 level voice messages (Ding Ding Ding), had a lower human evaluation of usability (*p* = 0.049 < 0.05) than Group 1 (*M* = 6.70, *SD* = 0.46). After back testing, satisfaction with time spent was significantly different between the groups (*p* = 0.01 < 0.05), while satisfaction with the level of difficulty (*p* = 0.374) and support information *(p* = 0.262) was not obviously different. It can be concluded that the disfluency of the task progress due to the lengthy voice in Task 3 triggered a lower usability evaluation of the participants in Group 2, and the additional information at the expense of time spent did not improve the usability evaluation in terms of the task difficulty and support information.

#### 5.2.3. Workload

**Figure 5:**
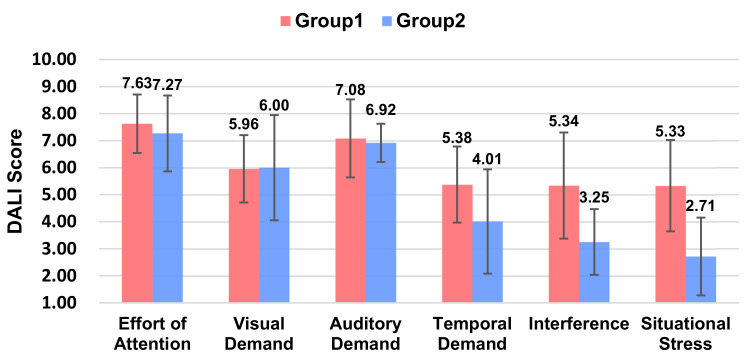
Ratings of the DALI scale between the two groups.

From the workload scores (Figure 5), it can be concluded that Group 2, i.e., with L1 level voice messages (Ding Ding Ding), participants had a lower workload (*p* = 0.002 < 0.01). In terms of the DALI detail scores, the main reasons were lower interference (*p* = 0.003 < 0.01) and lower situational stress (*p* = 0.002 < 0.01).

#### 5.2.4. Trust

Group 1 had similar trust scores (*M* = 6.43, *SD* = 0.67) to Group 2 (*M* = 6.48, *SD* = 0.51) with no significant difference (*p =* 0.519). The three dimensions of predictability (*p* = 0.392), dependability (*p* = 0.954), and desire to continue using (*p* = 0.717) did not produce distinct differences in terms of each of the trust scores.

#### 5.2.5. Sweep

**Figure 6:**
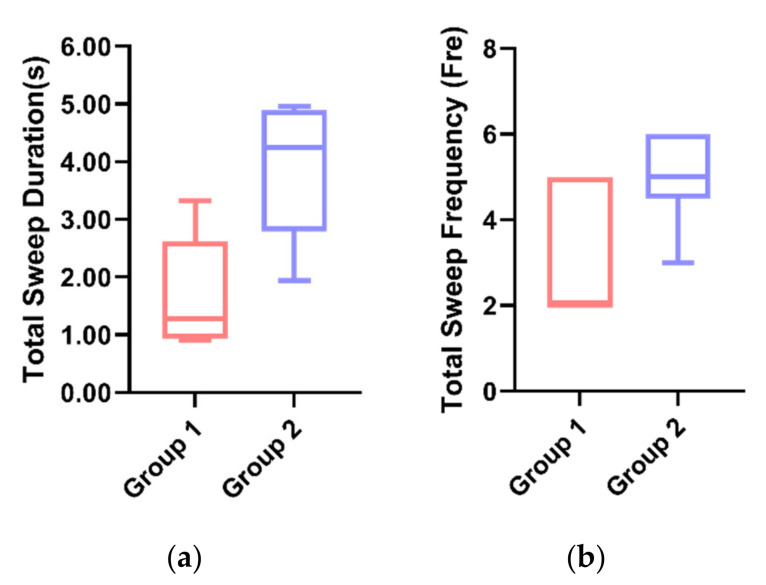
(**a**) Individual differences in the total sweep duration between the two groups. (**b**) Individual differences in the total sweep frequency between two groups.

In Task 3, the average sweep time (Figure 6a) was significantly lower in Group 1 (*M* = 1.671, *SD* = 0.39) than in Group 2 (*M* = 3.893, *SD* = 1.15) by the *t*-test (*p* = 0.00 < 0.01). Similarly, the sweep time means were also significantly lower in Group 1 than in Group 2 (*p* = 0.02 < 0.05) (Figure 6b), indicating that Group 1 enabled participants to focus more on the driving task and was less likely to pose a hazard to driving.

#### 5.2.6. Affect

**Figure 7:**
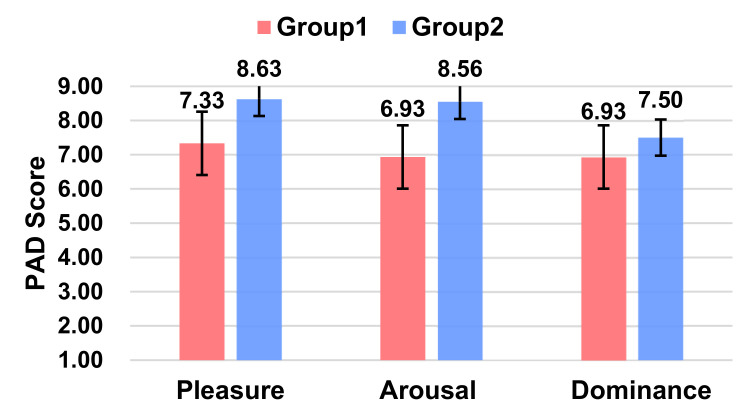
Ratings of Pleasure, Arousal, and Dominance between the two groups.

From the PAD scores (Figure 7), it can be concluded that Group 2, i.e., with L1 voice information (Ding Ding Ding), was able to make participants acquire more positive emotions, with higher levels of pleasure (*p* = 0.001 < 0.01) and activity (*p* = 0.001 < 0.01) and no significant difference in dominance (*p* = 0.202).

In conclusion, the L1 of voice information (Ding Ding Ding) added to Group 2 was able to make participants gain more positive emotions and reduce their workload. At the same time, participants rated the usability of Experiment 2 as lower. It is probably because the “Ding Ding Ding” voice had no obvious content meaning, which made the participants uncomfortable or did not have sufficient task fluency. Combining the vehicle driving data and the sweeping data, it was concluded that the participants in Group 1 had significantly better lateral control of the vehicle and a higher concentration on driving and had the best safety in terms of the vehicle safety dimensions.

### 5.3. Task Three: Chatting

#### 5.3.1. Safety

In Task 4, after the *t*-test, there was no significant difference in the average standard deviation of vehicle speed in Group 1 (*M* = 1.14, *SD* = 0.64) compared to Group 2 (*M* = 0.90, *SD* = 0.32). Moreover, the average standard deviation (*M* = 1.09, *SD* = 0.59) of Group 1 had no significant difference compared to Group 2 (*M* = 1.34, *SD* = 0.72) (*p* = 0.06 > 0.05), so that the driver was not considered to be very different in the two sets of vehicle controls.

#### 5.3.2. Usability

The participants in Group 2 rated higher overall than Group 1, but there was no significant difference (*p* = 0.355). As seen in the three usability scores, the difficulty satisfaction dimension was significantly different (*p* = 0.027 < 0.05), and the two dimensions of satisfaction with the time required to complete the tasks (*p* = 0.327) and satisfaction with support information (*p* = 0.659) did not produce significant differences. Thus, Group 1, where the robots have no expressions and movements, had lower difficulty satisfaction scores in the human evaluation of their usability, meaning that it would be harder to use robots without expressions and movements.

#### 5.3.3. Workload

**Figure 8:**
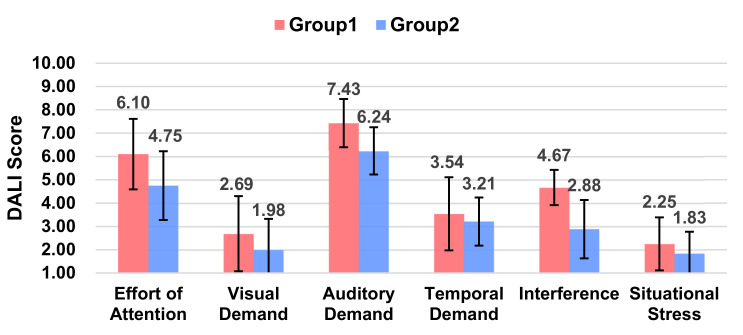
Ratings of Pleasure, Arousal, and Dominance between the two groups.

The overall average value of the workload was lower in Group 2 than in Group 1, but there is no significant difference (*p* = 0.495). As seen on each of the workload scores (Figure 8), Group 2 produced significant variability in both the auditory demand (*p* = 0.005 < 0.05) and interference (*p* = 0.000 < 0.001). That is, expressionless and voiceless robots will increase the auditory demand of the driver and cause stronger interference with the driver.

#### 5.3.4. Trust

There was no significant difference between the two groups in the mean trust score (*p* = 0.747). The dimensions of predictability (*p* = 0.281), dependability (*p* = 0.855), and desire to continue using (*p* = 0.422) did not produce significant differences in the scores of the trustworthiness dimensions.

#### 5.3.5. Sweep

**Figure 9:**
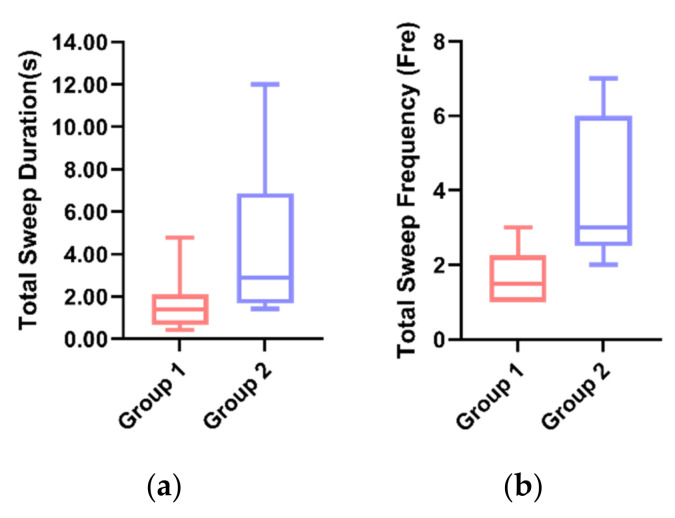
(**a**) Individual differences in the total sweep duration between the two groups. (**b**) Individual differences in the total sweep frequency between the two groups.

By the *t*-test, the average value of the total sweep duration for Group 1 (*M* = 1.695, *SD* = 1.40) was significantly lower (*p* = 0.01 < 0.05) than that of Group 2 (*M* = 4.439, *SD* = 1.83) (Figure 9a). Additionally, the mean of the total sweep frequency in Group 1 (*M* = 1.67, *SD* = 0.82) was significantly lower (*p* = 0.02 < 0.05) than in Group 2 (*M* = 4.00, *SD* = 1.94) (Figure 9b). The results indicated that the participants in Group 2 had a significantly higher sweep duration and sweep frequency than the participants in Group 2, which may be due to the participants’ tendency to receive eye contact from the robot with anthropomorphic visual expressions.

#### 5.3.6. Affect

**Figure 10:**
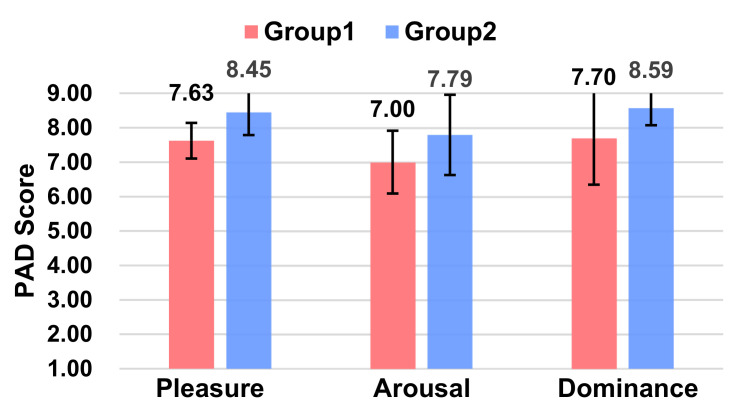
Ratings of Pleasure, Arousal, and Dominance between the two groups.

From the PAD scores (Figure 10), it can be concluded that Group 2, where the robot has anthropomorphic human expressions, was able to give the participants a higher level of pleasure score. The two experimental groups were significant only in terms of the pleasure score (*p* = 0.005 < 0.05), and no notable differences were seen in terms of both the arousal (*p* = 0.079) and dominance scores (*p* = 0.066).

In summary, compared to Group 1, Group 2 has added visual information, as long as the voice information was able to make the participants gain more positive emotions and a correspondingly moderate amount of attention, since the participants in Group 1 were communicated with via voice interactions, which led to a significantly higher auditory load on the participants and produced some interference. In addition, the participants’ ability to control the vehicle laterally was not as good as Group 2 with expressions, so the robot with expressions was better than the robot without expressions (pure voice). 

## 6. Discussion

Effective human resource information is a key component of human–robot team performances. Designing robots for human transparency is critical, particularly to promote the appropriate trust, workload, usability, affect, understanding of the robot, and ultimately, human–robot performance. Despite a growing amount of literature on transparency and how anthropomorphic humans can be applied to intelligent agents [11,21,22], the literature applying transparency principles to robotic communication is still far from adequate. This paper explores the application of the transparency models proposed by Chen et al. [10,11] to human–robot interactions in smart car scenarios in an attempt to test their impact on human–robot teams.

### 6.1. Transparency

The results of the study on the transparency of robot–human communication showed that, firstly, it is not that more transparency is better; secondly, the increase of information is related to the characteristics of the interaction channel. The information of the voice channel can accurately broadcast the intentions that the robot wants to express, but at the same time, it is important to pay attention to the accuracy and rapidity of the expression and the conciseness and clarity of the text content. The voice of the L3 level (predetermination) needs to be shown in a general situation. The L1 (perceptual) voice does not perform well in terms of usability performance and can be removed if the conversation is long. Information from the visual channel enhances the receptivity of the information, but given the fluency of the communication, not every level is suitable for display; otherwise, this would make the change of expression too cumbersome. If the communication is initiated by a human, then the visual information of the L1 listening state is necessary to effectively indicate that the robot is receiving the information from the human. If the communication is not human-initiated, the information can be directed to the L2 comprehension level, which allows the human to quickly accept the information expressed by the robot. Additionally, the visual information of L3 is suggested to be provided in order to improve the human’s pleasure, especially in the stationary state. In summary, the visual and acoustic information of L2 and L3 are suggested to be provided to the human for a good communication result; the visual information of L1 is not good for human–robot communication, except when the human initiates it.

### 6.2. Anthropomorphism

For robot anthropomorphism, the result is that the voice performance in L1 is poor, and the visual performance in L3 is good. The anthropomorphic sound information in L1 may disturb the driver while driving, showing that this will make the workload increase because of the disturbance, and the usability of the robot will be reduced accordingly, despite that it will make the driver more pleasant. In other words, the premise of not interfering with driving should be met first while pursuing a humanoid emotional performance.

The study also compared the visual channel. The robot with visual expressions was not better than the one without visual expressions in terms of driving performance, but it was better in terms of usability, because it was easier to use. Meanwhile, it increased the drivers’ positive emotions. Increasing the visual expression of L3 would also enhance the humanoid nature of the robot, gain more attention from the driver, and make it more emotionally pleased. Therefore, the anthropomorphism of sound was not confirmed in this study, but the anthropomorphism of expressions had a good performance.

### 6.3. HRI Variable Relationships

Finally, we observed the relationship between robots conveying information and safety, usability, workload, trust, and affect. In scenarios where the human and robot collaborate on secondary tasks (experimental phone calls, chats, and so on) and people focus their attention on driving with fewer sweeps, the robot usability is better, workload lower, and the human has better control of the vehicle. In the stationary state, people have more interest in the robot and a correspondingly better mood in the case of more appropriate sweep times.

### 6.4. Implications for HRI Design

Overall, the results of this study showed the complexity of designing transparent communication with robotically anthropomorphic two-channel audio–visual interactions. Transparent communication that conveys a robot’s state may be useful in terms of the human performance, but it may also be onerous in terms of the workload if the details of the application (e.g., text mode) are not considered. The text mode needs to be combined with other modes (e.g., graphics), and certain levels of transparency are more appropriate for the text mode, such as L2 and L3, due to the nature of the information available in L3 transparency. However, if the robot engaging in a passive interaction, then the graphical form is more suitable for L1 transparency. Conversely, if it is engaging in an active interaction, it does not need L1 and goes directly to L2. The use of a graphical form for L3 was not necessary but added to the emotional experience.

The information representation of robots is not a fixed pattern of information transparency; it has different applications in different types of scenarios, and experiments have found that what they have in common still remains to be discussed carefully by situation. In future robotic systems, these design trade-offs must be made.

### 6.5. Limitations of the Current Study

This study had several limitations. The first is that, due to the limitations of the experiment, only three scenarios were set up for the experiment, while the scenarios of the human–robot interactions were complex and diverse. Secondly, the results of this study were not necessarily fully applicable to all scenarios, such as dangerous driving-type scenarios. Our task scenarios focused on one round of interaction processes—namely, one round of human–robot communication for perception, understanding, and prediction. Additionally, there are many scenarios that require multiple rounds of communication. For example, when the robot helps navigate the whole process, whether it needs to comprehend and anticipate transparent information every time when confirming the navigation location, route, and time remains to be discussed separately.

## 7. Conclusions and Future Directions

Future research may involve exploring the use of tone words to convey level 1 anthropomorphic vocal information and overall utterance expressions and patterns of how to communicate emotionally with people. For example, when a call is about to come in, the robot’s voice of “Ding Ding Ding, Xiao Wang is calling you” is replaced with “Master, your friend Xiao Wang is calling”. This may convey an anthropomorphic and emotional tone in a shorter text, because the robot has a consistent personal address with the driver and understands the caller’s relationship with the driver. In addition, multiple rounds of human–robot interactions can continue to be explored for providing further research for more complex scenarios.

In summary, the current research applies an emerging human–robot team transparency model and a simple anthropomorphic approach to the way intelligent robots communicate. It also provides a reference for the design of human–robot interactions that can maintain good performances after empirical evaluations. The results suggest that higher transparency (L3 voice and vision) can improve the human–robot team performance and that lower levels of transparency (L1 voice) need not be demonstrated. The robot’s perceptual information when listening to a human (L1 vision) and robot understanding (L2 voice) are the fundamental conditions for communication between the two parties. The current study also showed that the robot anthropomorphic voice expression did not see a significant enhancement effect, even in terms of the user emotion enhancement, an aspect that requires more in-depth research and should not be limited to the level of voice interactions.

## Figures and Tables

**Table 1:** Summary table of the experimental results. The red font marks the better data that has been statistically tested. The reasons for choosing a better group are explained at the end of the table.

		Task 1: Welcome			Task 2: Incoming Call		Task 3: Chatting	
		Group 1	Group 2	Group 3	Group 1	Group 2	Group 1	Group 2
Transparency information		L2: voice and vision; L3: voice	L1: voice; L2: voice and vision; L3: voice	L1: voice; L2: voice and vision; L3: voice and vision	L2: voice and vision; L3: voice and vision	L1: voice; L2: voice and vision; L3: voice and vision	L2: voice; L3: voice	L1: vision; L2: voice; L3: voice and vision
① Safety	Standard Deviation of Vehicle Speed	/			0.42	0.27	1.14	0.90
	Standard Deviation of Left Lane Departure				0.18	0.77	1.09	1.34
② Usability	Total Means	/			6.70	6.32	6.17	6.45
	Ease of Task Completion				6.61	6.42	5.70	6.27
	Time Required to Complete Tasks				6.79	6.04	6.50	6.69
	Satisfaction with Support Information				6.71	6.50	6.80	6.40
③ Workload	Total Means	/			6.12	5.03	4.45	3.48
	Effort of Attention				7.63	7.27	6.10	4.75
	Visual Demand				5.96	6.00	2.69	1.98
	Auditory Demand				7.08	6.92	7.43	6.24
	Temporal Demand				5.38	4.01	3.54	3.21
	Interference				5.34	3.25	4.67	2.88
	Situational Stress				5.33	2.71	2.25	1.83
④ Trust	Total Means	/			6.43	6.48	6.33	6.35
	Predictability				6.13	6.36	6.08	6.39
	Dependability				6.59	6.57	6.36	6.30
	Loyalty/Desire to continue using				6.57	6.50	6.55	6.35
⑤ Sweep	Total Sweep Duration(s)	1.973	4.648	4.015	1.671	3.893	1.695	4.439
	Total Sweep Frequency (Fre)	2.50	2.50	2.67	3.00	5.00	1.67	4.00
⑥ Affect	Pleasure Score	7.37	7.25	7.70	7.83	8.63	7.63	8.45
	Arousal Score	7.60	7.29	6.33	6.93	8.56	7.00	7.79
	Dominance Score	8.50	8.04	8.50	6.93	7.50	7.70	8.59
Conclusion		The better group: Group 3 Reason: L1 anthropomorphic voice information and L3 level visual information can get more attention from the participants and, at the same time, obtain a more positive emotional experience.			The better group: Group 1 Reason: Adding L1 level voice information (Ding Ding Ding), Group 2 can make the participants have more positive emotions and reduce the workload. However, the evaluation of the usability was lower, possibly because the voice of “Ding Ding Ding” made people feel uncomfortable. At the same time, the participants in Group 1 had significantly better vehicle lateral control levels and better safety.		The better group: Group 2 Reason: Compared with Group 1, Group 2 added visual information that can make the participants have more positive emotions. Group 1 communicated with the participants only through voice interactions, which caused the participants’ temporal loads to be significantly higher and caused certain interferences. Additionally, the ability of Group 1 to control the vehicle laterally was not as good as Group 2, so the robots with expressions were better than a pure voice robot.	

## Data Availability

Data sharing is not applicable.
